# The quality of severe mental disorder diagnoses in a national health registry as compared to research diagnoses based on structured interview

**DOI:** 10.1186/s12888-017-1256-8

**Published:** 2017-03-14

**Authors:** Ragnar Nesvåg, Erik G. Jönsson, Inger Johanne Bakken, Gun Peggy Knudsen, Thomas D. Bjella, Ted Reichborn-Kjennerud, Ingrid Melle, Ole A. Andreassen

**Affiliations:** 1 Norwegian Institute of Public Health, P.O. Box 4044, Nydalen, N-0403 Oslo, Norway; 2 Norwegian Center for Mental Health Research (NORMENT), Oslo University Hospital & University of Oslo, P.O. Box 4956, Nydalen, N-0424 Oslo, Norway; 3 Department of Clinical Neuroscience, Centre of Psychiatric Research, Karolinska Institutet, Administration, Tomtebodavägen 18A, 5th floor, 171 77 Stockholm, Sweden; 4 Institute of Clinical Medicine, University of Oslo, P.O. Box 1171, Blindern, N-0319 Oslo, Norway

**Keywords:** Psychosis, Schizophrenia, Bipolar disorder, Registry, ICD-10, DSM-IV

## Abstract

**Background:**

Utilization of diagnostic information from national patient registries rests on the quality of the registered diagnoses. We aimed to investigate the agreement and consistency of diagnoses of psychotic and bipolar disorders in the Norwegian Patient Registry (NPR) compared to structured interview-based diagnoses given as part of a clinical research project.

**Methods:**

Diagnostic data from NPR were obtained for the period 01.01.2008–31.12.2013 for all patients who had been included in the Thematically Organized Psychosis (TOP) study between 18.10.2002 and 01.09.2014 with a Diagnostic and Statistical Manual of Mental Disorders, 4th edition (DSM-IV) diagnosis of schizophrenia (*n* = 537), delusional disorder (*n* = 48), schizoaffective disorder (*n* = 118) or bipolar disorder (*n* = 408). Diagnostic agreement between the primary DSM-IV diagnosis in TOP and the International Classification of Diseases, 10th revision (ICD-10) diagnoses in NPR was evaluated using Cohen’s unweighted nominal kappa (κ). Diagnostic consistency was calculated as the proportion of all registered severe mental disorder diagnoses in NPR that were equivalent to the primary diagnosis given in the TOP study.

**Results:**

The proportion of patients registered with the equivalent ICD-10 diagnosis as the primary DSM-IV diagnosis given in TOP was 84.2% for the schizophrenia group, 68.8% for the delusional disorder group, 76.3% for the schizoaffective disorder group, and 78.4% for the bipolar disorder group. Diagnostic agreement was good for schizophrenia (κ = 0.74) and bipolar disorder (κ = 0.72), fair for schizoaffective disorder (κ = 0.63), and poor for delusional disorder (κ = 0.39). Among patients with DSM-IV schizophrenia, 4.7% were diagnosed with ICD-10 bipolar disorder, and among patients with DSM-IV bipolar disorder, 2.5% were diagnosed with ICD-10 schizophrenia. Diagnostic consistency was 84.9% for schizophrenia, 59.1% for delusional disorder, 65.9% for schizoaffective disorder, and 91.0% for bipolar disorder.

**Conclusions:**

When compared to research-based diagnoses, clinical diagnoses of schizophrenia and bipolar disorder in the NPR are accurate and consistent, with minimal diagnostic overlap between the two disorders.

## Background

Data from national health registries represent a unique source for research into health service quality and disease epidemiology. Population-based health care registry data are particularly important for rare and severe diseases like psychotic disorders due to complete national coverage. When using such data, information about diagnostic accuracy and consistency is crucial. A review of studies evaluating diagnostic information in the Swedish National Inpatient Register concluded that the validity was high for most, but not all types of somatic and psychiatric diagnoses [1]. Systematic assessments of diagnostic accuracy in health registries in Denmark [2], Sweden [3, 4], and Finland [5–7] have generally shown good agreement between register diagnoses of schizophrenia spectrum disorders and research diagnoses based on case notes and/or structured diagnostic interviews.

In industrialized countries, the great majority of people with severe mental disorders will be in contact with public health care facilities at some point during their lifetime. Data from Israel indicate that 93% of patients diagnosed with schizophrenia in an epidemiological survey were also registered with schizophrenia in a national psychiatric registry [8].

The Norwegian Patient Registry (NPR) includes subject-specific administrative and diagnostic information from 2008 and onwards from all governmentally funded specialist health treatment facilities, i.e. public hospitals, private clinics funded by the government, and private specialists who receive reimbursement for their treatment of patients. This includes the majority of all psychiatric treatment in Norway, as there are no psychiatric hospitals without government support, and only few fully private psychiatrists. In NPR the diagnoses are registered as International Classification of Diseases, 10th revision (ICD-10 [9]) codes as reported by the treating clinicians. In psychiatric care settings, diagnoses are typically determined by the physician or psychologist, either alone or after a discussion with colleagues and staff members. For psychiatric disorders, data from the NPR have previously been used to investigate prevalence of neurodevelopmental disorders among children [10], co-morbid substance use disorders in severe mental disorders [11], and prevalence of self-poisoning with medications among adolescents [12]. The quality of mental disorder diagnoses in the NPR is at present unknown.

The aim of the present study was to investigate the quality of severe mental disorder diagnoses (schizophrenia, delusional disorder, schizoaffective disorder, and bipolar disorder) in the NPR by comparing diagnoses based on structured diagnostic interview and review of case notes as part of a clinical research project with diagnoses given by the treating clinicians and reported to the NPR. We specifically aimed to investigate diagnostic agreement and consistency. Our hypothesis was that a schizophrenia diagnosis would show better agreement than schizoaffective and bipolar disorder diagnoses.

## Methods

### Data sources

The study sample consisted of 1111 patients who were recruited to the Thematically Organized Psychosis (TOP) study between 18.10.2002 and 01.09.2014 and had consented to registry linkage. The TOP study was initiated at the University of Oslo in 2002 and is an ongoing multicenter, multidisciplinary investigation of clinical, genetic, neuroimaging, pharmacological and neurocognitive features of schizophrenia and bipolar disorders. Details regarding recruitment of patients and clinical procedures have been reported previously [13]. Briefly, patients with psychotic disorders were consecutively recruited from the major hospitals in the Oslo region and clinically assessed by trained research physicians, psychiatrists, and psychologists. All patients were diagnosed according to the Diagnostic and Statistical Manual of Mental Disorders, 4th edition (DSM-IV [14]) following a semi-structured interview [15] and review of case notes. The diagnostic assessment in the TOP study has very good reliability, and the overall agreement for the DSM-IV diagnostic categories tested is 82% and the overall Kappa 0.77 (95% CI, 0.60–0.94) [13].

For the present study only the main diagnosis at the patients’ first participation in the TOP study was used for comparison with registry data. The patients were grouped into four diagnostic categories, based on their primary DSM-IV diagnosis at inclusion to the TOP study: schizophrenia (including schizophreniform disorder) (*n* = 537), delusional disorder (*n* = 48), schizoaffective disorder (*n* = 118), and bipolar disorder (including bipolar disorder, type I, bipolar disorder, type II, and bipolar disorder, not otherwise specified) (*n* = 408).

The NPR is a national health registry covering all sectors of the governmentally funded specialized health care services in Norway, including somatic, psychiatric and substance use treatment facilities. All hospitals, clinics and private practitioners who receive governmental reimbursement are obliged to report activity and diagnostic data to the NPR. In Norway, there are publicly funded mental health care facilities for in- and outpatient treatment in all regions of the country. There is a maximum annual fee for outpatient treatment (in 2016: 2185 NOK, appr. 230 €) while inpatient treatment is free of charge.

The unique 11-digit personal identification number has been reported for each patient to the NPR from 2008 onwards. This allows for linkage with other data sources as well as keeping track of individual disease trajectories across treatment facilities in Norway.

For the present study, data from NPR for the period 01.01.2008–31.12.2013 were obtained for all patients within the four diagnostic categories defined above who had been recruited to TOP and consented to registry linkage. Data on hospital stays and outpatient consultations were retrieved from the NPR. All available NPR data were used irrespective of when the patient was included in the TOP study. We had information on the level of care (inpatient/outpatient), type of care (somatic hospital, mental health care facility, substance use treatment facility or psychiatrists/psychologists with governmental reimbursement), and assigned ICD-10 diagnoses. All ICD-10 codes for mental disorders (F00–F99) were included. Specifically, the study looked into four major severe mental disorder categories: schizophrenia (F20), persistent delusional disorder (F22), schizoaffective disorder (F25), and bipolar disorder (F30–F31). Additionally, to calculate diagnostic agreement and consistency, for the four main diagnostic categories, a broader group of «severe mental disorders» was formed which included schizophrenia-like psychotic disorder (F20–F29), bipolar disorder (F30–F31), and depressive disorder with psychotic features (F32.3, F33.3). To determine the number of specialist health care contacts, which included a psychiatric disorder diagnosis, a «mental disorder» category was formed, which included all F-codes in the ICD-10 (F00–F99).

### Statistical Analysis

Differences in demographic and clinical data between the four diagnostic groups were analyzed using Kruskall-Wallis equality-of-populations rank test with Dunn’s post-hoc pairwise comparison. The number of registrations in the NPR with each of the selected ICD-10 categories was calculated for each of the four DSM-IV diagnostic groups.

Diagnostic agreement was calculated as the proportion of patients who were either correctly classified as having the diagnosis in question (schizophrenia, delusional disorder, schizoaffective disorder, or bipolar disorder), or correctly classified as not having the diagnosis in question. The precision of diagnoses in NPR compared to TOP was further evaluated using Cohen’s unweighted nominal kappa (κ), a metric that estimates overall agreement while taking into account the possibility of the agreement occurring by chance [16]. To evaluate the predictive properties of severe mental disorder diagnoses in NPR, sensitivity (i.e. the probability that an ill person will receive the correct diagnosis), specificity (i.e. the probability that a person without the illness will not receive the diagnosis), positive predictive value (PPV, i.e. the probability that a person diagnosed as ill is truly ill) and negative predictive value (NPV, i.e. the probability that a person not registered with the disorder truly did not have the disorder) were calculated. In the analyses of agreement, kappa, sensitivity, specificity, PPV, and NPV, the total group of 1111 patients were considered to be the target population, patients within one of the four DSM-IV diagnostic categories under scrutiny were considered to be truly ill, while patients with the remaining three diagnostic categories were considered to be “not ill”. The occurrence of the equivalent ICD-10 diagnostic category at least once in the NPR was considered to be a positive test, while absence of registrations in NPR with the equivalent ICD-10 diagnostic category was considered to be a negative test.

Diagnostic consistency was calculated as the percentage of all registered contacts in NPR with a severe mental disorder diagnosis (i.e. ICD-10 codes F20–F29, F30–F31, F32.3, or F33.3) that included the primary DSM-IV diagnosis assigned when recruited to the TOP-study.

To investigate if diagnostic agreement or consistency differed between sexes, we performed supplementary analyses of men and women separately. Since patients were usually recruited to the TOP study while in treatment in specialized health care, the diagnostic data from the TOP study and the NPR are not completely independent. The degree of dependency is presumably higher for patients recruited during the period NPR-data were available (2008–2013) than for patients recruited prior to this period. To test if time of recruitment affected the estimates of diagnostic agreement and consistency, we performed supplementary analyses after splitting the sample into two groups: patients recruited to TOP before NPR-data were available, i.e. before 01.01.2008, and patients recruited when subject-specific NPR-data were available, i.e. after 01.01.2008. All statistical analyses were performed in STATA 14 (StataCorp. 2015. Stata Statistical Software: Release 14. College Station, TX: StataCorp LP.)

## Results

### Demographic and clinical data

Demographic and clinical data for the 1111 patients are presented in Table 1. There were significantly more men in the schizophrenia and delusional disorder groups compared to the schizoaffective and bipolar disorder groups. Mean age at inclusion to the TOP study ranged between 30.3 years for the schizophrenia group to 35.4 years for the delusional disorder group. Patients with schizophrenia or schizoaffective disorder had significantly more registrations with any or severe mental disorders, more hospitalizations, outpatient visits and bed-days compared to patients with delusional disorder or bipolar disorder.

**Table 1:** Demographic and clinical data for 1111 patients recruited to the Thematically Organized Psychosis (TOP) Study

TOP-diagnosis (DSM-IV)	SCZ (*n* = 537)		DEL (*n* = 48)		SCA (*n* = 118)		BIP (*n* = 408)		test (*p*-value)^a^
% men	63.5		64.6		35.6		40.0		.0001^b^
Age (mean, SD)	30.3	9.4	35.4	9.8	32.3	11.2	34.5	12.1	.0001^c^
	Median	IQR	Median	IQR	Median	IQR	Median	IQR	
Months of NPR data pre TOP	2	0–21	10	0–40	2	0–21	0	0–17	.0935
Months of NPR post TOP	69	49–39	61	31–74	66	37–99	71	54–96	.0365^d^
Any mental disorder (ICD-10 F00–99)	52	21–106	27	10–79	46	16–90	26	5–61	.0001^e^
Severe mental disorder (ICD-10 F20–F31, F32.3, F33.3)	45	13–97	16	4–45	37	10–76	14	1–45	.0001^f^
Hospitalizations	3	1–7	1	0–3	4	1–9	2	0–5	.0001^g^
Outpatient visits	72	34–137	48	28–90	74	32–115	43	16–84	.0001^h^
Bed-days	87	2–330	11	0–91	61	4–257	10	0–79	.0001^i^

*Abbreviations*: *DSM-IV* Diagnostic and Statistical Manual of Mental Disorders, 4th edition, *SCZ* schizophrenia, *DEL* delusional disorder, *SCA* schizoaffective disorder, *BIP* bipolar disorder (type I, type II, and bipolar disorder not otherwise specified), *SD* standard deviation, *IQR* interquartile range, *NPR* Norwegian Patient Registry, *ICD-10* International Classification of Diseases 10th revision

^a^ Kruskall-Wallis equality-of-populations rank test with Dunn’s post-hoc pairwise comparison

^b^ *χ* ^2^ = 50.8 (SCZ > SCA, *p* < 0.0001; SCZ > BIP, *p* < 0.0001; DEL > SCA, *p* = 0.0004; DEL > BIP, *p* = 0.0005)

^c^ *χ* ^2^ = 31.9 (DEL > SCZ, *p* = 0.0004; DEL > SCA, *p* = 0.02; BIP > SCZ, *p* < 0.0001; BIP > SCA, *p* = 0.03)

^d^ *χ* ^2^ = 8.5 (SCZ > DEL, *p* = 0.009; SCA > DEL, *p* = 0.01; BIP > DEL, *p* = 0.002)

^e^ *χ* ^2^ = 66.4 (SCZ > DEL, *p* = 0.002; SCZ > BIP, *p* < 0.0001; SCA > DEL, *p* = 0.0411; SCA > DEL, *p* = 0.0001)

^f^ *χ* ^2^ = 85.2 (SCZ > DEL, *p* = 0.0003; SCZ > BIP, *p* < 0.0001; SCA > DEL, *p* = 0.01; SCA > BIP, *p* < 0.0001)

^g^ *χ* ^2^ = 35.3 (SCZ > DEL, *p* = 0.0001; SCZ > BIP, *p* < 0.0001; SCA > DEL, *p* = 0.0001; SCA > BIP, *p* < 0.0001)

^h^ *χ* ^2^ = 64.9 (SCZ > DEL, *p* = 0.02; SCZ > BIP, *p* < 0.0001; SCA > DEL, *p* = 0.04; SCA > BIP, *p* < 0.0001)

^i^ *χ* ^2^ = 71.6 (SCZ > DEL, *p* = 0.0001; SCZ > BIP, *p* < 0.0001; SCA > DEL, *p* = 0.001; SCA > BIP, *p* < 0.0001)

### Diagnostic agreement

The proportion of patients registered in NPR with the equivalent ICD-10 diagnosis as the primary DSM-IV diagnosis given in TOP was 84.2% for the schizophrenia group, 68.8% for the delusional disorder group, 76.3% for the schizoaffective disorder group, and 78.4% for the bipolar disorder group (Table 2). In the schizophrenia group, 7.1% had received an ICD-10 diagnosis of schizoaffective disorder, and 4.7% had been diagnosed with bipolar disorder in the NPR. Conversely, in the bipolar disorder group, 2.5% had ever received an ICD-10 schizophrenia diagnosis, and 2.7% a persistent delusional disorder diagnosis in NPR (Table 2). Kappa reliability measures were higher for schizophrenia (0.74) and bipolar disorder (0.72) than for schizoaffective (0.63) and delusional disorder (0.39) (Table 3). Diagnostic specificity was high (0.90–0.94) for all diagnostic categories. PPV was high for schizophrenia and bipolar disorder (0.88 and 0.86, respectively), moderate for schizoaffective disorder (0.61), and low for delusional disorder (0.31). NPV was high (0.86–0.97) for all diagnostic categories.

**Table 2:** Concordance^a^ between ICD-10 diagnoses in the Norwegian Patient Registry (NPR) and DSM-IV diagnosis in the Thematically Organized Psychosis (TOP) study

TOP group		SCZ (*n* = 537)		DEL (*n* = 48)		SCA (*n* = 118)		BIP (*n* = 408)	
*NPR diagnoses*	*ICD-10-codes*	*n*	%	*n*	%	*n*	%	*n*	%
Schizophrenia	F20	452	84.2	9	18.8	40	33.9	10	2.5
Delusional disorder	F22	54	10.1	33	68.8	9	7.6	11	2.7
Schizoaffective disorder	F25	38	7.1	0	0	90	76.3	20	4.9
Bipolar disorder	F30–F31	25	4.7	5	10.4	24	20.3	320	78.4

Data obtained from NPR for the period 2008–2013 on patients recruited to TOP between 18.10.2002 and 01.09.2014

*Abbreviations*: *ICD-10*, International Classification of Diseases, 10th revision, *DSM-IV* Diagnostic and Statistical Manual of Mental Disorders, 4th edition, *SCZ* schizophrenia, *DEL* delusional disorder, *SCA* schizoaffective disorder, *BIP* bipolar disorder (type I, type II, and bipolar disorder not otherwise specified)

^a^ Percentage of patients within each of the four TOP groups that were registered with each of the four equivalent ICD-10 categories at least once in the NPR during 2008–2013

**Table 3:** Agreement statistics between ICD-10 diagnoses in the Norwegian Patient Registry (NPR) and primary diagnoses according to DSM-IV in the Thematically Organized Psychosis (TOP) study

TOP-group		Agreement^a^		Kappa		Sensitivity		Specificity		PPV		NPV	
		Est.	95% CI	Est.	95% CI	Est.	95% CI	Est.	95% CI	Est.	95% CI	Est.	95% CI
SCZ	All	.87	.85–.89	.74	.68–.80	.84	.81–.87	.90	.87–.92	.88	.86–.91	.86	.83–.89
	Men	.85	.82–.88	.69	.61–.77	.84	.81–.88	.86	.81–.90	.89	.86–.93	.79	.74–.84
	Women	.89	.87–.92	.77	.68–.85	.84	.78–.89	.93	.90–.95	.87	.82–.92	.91	.88–.94
	Pre NPR^b^	.88	.85–.91	.76	.67–.84	.85	.81–.90	.91	.87–.94	.89	.85–.93	.87	.83–.91
	Post NPR^c^	.86	.83–.89	.72	.63–.81	.83	.79–.88	.89	.85–.93	.88	.84–.92	.85	.81–.89
DEL	All	.92	.90–.93	.39	.34–.44	.69	.55–.82	.93	.92–.95	.31	.22–.40	.99	.98–.99
	Men	.90	.88–.92	.35	.27–.42	.61	.43–.79	.92	.89–.94	.29	.18–.41	.98	.96–.99
	Women	.94	.92–.96	.45	.37–.53	.82	.62–1.0	.95	.93–.97	.33	.18–.48	.99	.99–1.0
	Pre NPR^b^	.94	.92–.96	.32	.24–.39	.57	.27–.87	.95	.93–.97	.24	.09–.40	.99	.98–1.0
	Post NPR^c^	.90	.88–.93	.42	.34–.49	.74	.58–.89	.91	.89–.93	.34	.23–.45	.98	.97–.99
SCA	Total	.92	.91–.94	.63	.57–.69	.76	.68–.84	.94	.93–.96	.61	.53–.69	.97	.96–.98
	Men	.93	.91–.95	.55	.47–.63	.69	.54–.84	.95	.93–.97	.51	.38–.64	.98	.96–.99
	women	.92	.89–.94	.68	.60–.77	.80	.71–.89	.93	.91–.96	.67	.57–.77	.97	.95–.98
	Pre NPR^b^	.91	.89–.94	.59	.51–.68	.73	.61–.85	.93	.91–.96	.57	.45–.69	.97	.95–.98
	Post NPR^c^	.93	.91–.95	.67	.59–.75	.79	.69–.90	.95	.93–.97	.64	.53–.75	.97	.96–.99
BIP	Total	.87	.85–.89	.72	.66–.78	.78	.74–.82	.92	.90–.94	.86	.82–.89	.88	.86–.90
	Men	.90	.87–.92	.74	.66–.82	.78	.71–.84	.94	.92–.96	.84	.78–.90	.92	.89–.94
	women	.85	.82–.88	.69	.60–.77	.79	.73–.84	.90	.86–.93	.87	.82–.91	.83	.79–.87
	Pre NPR^b^	.86	.83–.89	.68	.60–.77	.72	.66–.78	.94	.91–.96	.88	.83–.93	.84	.81–.88
	Post NPR^c^	.89	.86–.91	.76	.67–.84	.85	.80–.90	.91	.88–.94	.84	.78–.89	.92	.89–.95

Data obtained from NPR for the period 2008–2013 on patients recruited to TOP between 18.10.2002 and 01.09.2014

*Abbreviations*: *ICD-10* International Classification of Diseases, 10th revision, *DSM-IV* Diagnostic and Statistical Manual for Mental Disorders, 4th edition, *PPV* positive predictive value, *NPV* negative predictive value, *Est.* estimate, *CI * confidence interval, *SCZ* schizophrenia, *DEL* delusional disorder, *SCA* schizoaffective disorder, *BIP* bipolar disorder

^a^ Proportion of patients who were either correctly classified as having the diagnosis in question, or correctly classified as not having the diagnosis in question

^b^ Patients included in TOP between 18.10.2002 and 31.12.2007

^c^ Patients included in TOP between 01.01.2008 and 01.09.2014

### Diagnostic agreement across gender and time of recruitment

For schizophrenia and bipolar disorder, the diagnostic agreement was similar in men and women, while for delusional and schizoaffective disorder the sensitivity was higher among women than men (Table 3). Kappa was higher for men than women in all diagnostic categories except for bipolar disorder, where women had higher kappa than men. Kappa was higher for schizophrenia patients who were recruited prior to 2008 compared to schizophrenia patients recruited in 2008 or later, while for the remaining diagnostic groups, kappa was higher among patients recruited in 2008 or later. Schizophrenia had high diagnostic sensitivity irrespective of time of inclusion, while for the remaining three categories the diagnostic sensitivity was higher for patients recruited to TOP concurrently with available NPR-data, i.e. after 01.01.2008, compared to patients recruited in the period 2002–2007. The diagnostic specificity was about 90% for all diagnostic categories with minimal differences across gender and time of inclusion. For schizophrenia and bipolar disorder, PPV was high across gender and time of recruitment, while for delusional disorder and schizoaffective disorder, PPV was higher among women than men, and higher among patients recruited in 2008 or later compared to patients recruited prior to 2008.

### Diagnostic consistency

Among the 537 patients in the schizophrenia group, 31,040 out of a total of 36,544 registered contacts with an ICD-10 severe mental disorder diagnosis in the NPR between 2008 and 2013 included a diagnosis of schizophrenia, constituting a diagnostic consistency of 84.9% (Table 4). Diagnostic consistency was 59.1% in the delusional disorder group, 65.9% in the schizoaffective disorder group, and 91.0% in the bipolar disorder group. In the schizophrenia group, 3.1% of the severe mental disorder contacts included a diagnosis of persistent delusional disorder, 4.1% included a diagnosis of schizoaffective disorder, and 1.6% included a diagnosis of bipolar disorder. In the bipolar disorder group, 1.3% of the severe mental disorder contacts included a diagnosis of schizophrenia, 1.1% included a diagnosis of persistent delusional disorder, and 4.1% included a diagnosis of schizoaffective disorder. Diagnostic consistency for schizophrenia was higher for men (87.5%) than for women (80.6%), and diagnostic consistency for delusional disorder was higher for women (72.8%) than for men (53.0%) (Table 5). For schizoaffective disorder and bipolar disorder the diagnostic consistency was not different between men and women. The diagnostic consistency was higher in the group of patients included to TOP prior to than after January 1st 2008 for all diagnostic groups (Table 5).

**Table 4:** Consistency^a^ between ICD-10 diagnoses in the Norwegian Patient Registry (NPR) and DSM-IV diagnosis in the Thematically Organized Psychosis (TOP) study

TOP group		SCZ (*n* = 537)		DEL (*n* = 48)		SCA (*n* = 118)		BIP (*n* = 408)	
*NPR diagnoses*	*ICD-10 codes*	*n*	%	*n*	%	*n*	%	*n*	%
Severe mental disorder	F20-F31, F32.3, F33.3	36,544	100	1661	100	6322	100	13,238	100
Schizophrenia	F20	31,040	84.9	523	31.5	1040	16.5	174	1.3
Delusional disorder	F22	1123	3.1	982	59.1	186	2.9	146	1.1
Schizoaffective disorder	F25	1509	4.1	0	0	4168	65.9	543	4.1
Bipolar disorder	F30–F31	599	1.6	66	4.0	695	11.0	12,044	91.0

*Abbreviations*: *ICD-10* International Classification of Diseases, 10th revision, *DSM-IV* Diagnostic and Statistical Manual of Mental Disorders, 4th edition, *TOP* Thematically Organized Psychosis, *SCZ* schizophrenia, *DEL* delusional disorder, *SCA* schizoaffective disorder, *BIP* bipolar disorder (type I, type II, and bipolar disorder not otherwise specified)

^a^ Percentage of all registered contacts with a severe mental disorder diagnosis (F20–F31, F32.3, F33.3) in the NPR during 2008–2013 that included the primary DSM-IV diagnosis assigned when recruited to the TOP-study between 18.10.2002 and 01.09.2014

**Table 5:** Consistency (%)^a^ between ICD-10 diagnoses in the Norwegian Patient Registry (NPR) and DSM-IV diagnosis in the Thematically Organized Psychosis (TOP) study across gender and time

	SCZ	DEL	SCA	BIP
All patients (*n* = 1111)	84.9	59.1	65.9	91.0
Men (*n* = 577)	87.5	53.0	65.1	92.6
Women (*n* = 534)	80.6	72.8	66.4	90.0
Patients included in TOP between 18.10.2002 and 31.12.2007 (*n* = 530)	88.5	87.1	75.9	93.5
Patients included in TOP between 01.01.2008 and 01.09.2014 (*n* = 581)	81.2	52.3	56.2	89.4

Data obtained from NPR for the period 2008–2013 on patients recruited to TOP between 18.10.2002 and 01.09.2014

*Abbreviations*: *ICD-10* International Classification of Diseases, 10th revision, *DSM-IV* Diagnostic and Statistical Manual of Mental Disorders, 4th edition, *SCZ* schizophrenia, *DEL* delusional disorder, *SCA* schizoaffective disorder, *BIP* bipolar disorder (type I, type II, and bipolar disorder not otherwise specified)

^a^ Percentage of all registered contacts with a severe mental disorder diagnosis (F20-F31, F32.3, F33.3) in the NPR that included the primary DSM-IV diagnosis assigned when recruited to the TOP-study

## Discussion

Among 1111 men and women with a diagnosis of schizophrenia, delusional disorder, schizoaffective disorder, or bipolar disorder, we found high degree of agreement between DSM-IV diagnoses as determined by structured diagnostic interview in a clinical research setting and ICD-10 diagnoses as determined by the treating clinicians and registered in the national patient registry. For schizophrenia and bipolar disorder, eight out of ten patients received the equivalent ICD-10 and DSM-IV diagnoses, with minimal differences between men and women. For bipolar disorder, diagnostic agreement was better for patients recruited in 2008 or later, while for schizophrenia the agreement was similar across time. For delusional and schizoaffective disorder patients the proportion of correctly diagnosed patients in the NPR ranged between 57 and 80%, with higher proportion for women compared to men, and higher proportion for patients recruited to the TOP study during the time NPR-data was available at individual level (2008–2013) compared to the period for which NPR data was not available (2002–2007). Poorer agreement among patients recruited prior to 2008 may be explained by some patients being lost to follow-up because they did not need treatment in specialist health care after inclusion.

The results indicate that diagnostic data from NPR show good agreement for severe mental disorders in general, but the level of agreement was best for schizophrenia (kappa 0.74) and bipolar disorder (kappa 0.72), fair for schizoaffective disorder (kappa 0.63), and poor for delusional disorder (kappa 0.39). Validity studies of registry data in other Nordic countries have also shown good agreement for schizophrenia [2–7, 17], but somewhat poorer agreement for schizoaffective disorder [2]. The poorer agreement for delusional and schizoaffective disorder in the current study may be explained by the low prevalence of the disorders in the TOP sample. Kappa reliability is dependent on prevalence of the disorder in addition to sensitivity and specificity [18]. Poorer agreement for schizoaffective disorder than for schizophrenia may also be explained by inherent differences in diagnostic criteria between ICD-10 and DSM-IV.

Few studies have investigated the validity of bipolar disorder diagnoses. In a previous study from Northern Norway, Øiesvold and colleagues demonstrated that diagnostic concordance for bipolar disorders was moderate (kappa ranging from 0.41 to 0.47) based on expert diagnostic interviews of a sample of 250 first-time admitted patients at a regional hospital [19]. Their study demonstrated higher concordance for manic episode than for bipolar depression, which indicates higher validity for bipolar disorder type I than bipolar disorder type II diagnoses in the NPR. In comparison, we found close to 80% agreement (kappa 0.72) for the total bipolar disorder group.

Patients diagnosed with schizophrenia or bipolar disorder based on a structured interview and review of case notes received the corresponding diagnosis at 85 and 91%, respectively, of registered contacts for severe mental disorders. Moreover, there were minimal overlap between schizophrenia and bipolar disorder, i.e. for schizophrenia only a few percent of the registered contacts included a diagnosis of bipolar disorder and vice versa. Hence the findings indicate that clinicians are consistent in their differentiation between schizophrenia spectrum disorders and bipolar disorders.

### Strengths and limitations

The major strength of this study lies in the high number of rigorously assessed patients recruited from several hospitals during a period of 12 years. Since the TOP-study was initiated several years before subject-specific data were available from the NPR the linked dataset allowed for study of time-sensitive agreement rates. However, the results must be interpreted with the following limitations in mind. Firstly, all patients in the TOP-study were recruited from hospitals and outpatient clinics, and the TOP interviewers were not blinded for clinical diagnoses. After inclusion the referring clinician received a written report which included diagnostic information. Thus, for most patients the two diagnostic assessments were not independent, which may have inflated the diagnostic agreement and consistency estimates. However, by investigating differences in diagnostic agreement and consistency across time, we were able to evaluate the putative time-dependent effect of this dependency. For the major diagnostic categories schizophrenia and bipolar disorder, there were only minor differences in agreement across time. Secondly, while the diagnoses in TOP are given according to DSM-IV, diagnoses in NPR are given according to ICD-10. Due to structural differences in the delineation of bipolar disorder between DSM-IV and ICD-10, we were not able to distinguish between bipolar disorder type I and type II in the present study. However, our findings indicate a high degree of accuracy for bipolar disorder diagnoses in general in the NPR. Thirdly, since only patients recruited to the TOP study were included in the present study, the results are not necessarily representative for all patients diagnosed with severe mental disorders in the NPR.

## Conclusions

More than eight out of ten patients diagnosed with schizophrenia or bipolar disorder in a clinical research project received the same diagnosis by their treating clinician as registered in the NPR. Moreover, the diagnostic consistency, i.e. proportion of severe mental disorder registrations in NPR with the equivalent ICD-10 diagnosis as the primary DSM-IV diagnosis given in the TOP-study, was 85% for schizophrenia and 91% for bipolar disorder. There were minimal differences in diagnostic agreement and consistency between genders and across time, and negligible diagnostic overlap between affective and non-affective disorders. The results support the use of registry-based diagnoses of schizophrenia and bipolar disorder when searching for cases in epidemiological and genetic studies involving severe mental disorders.

## Abbreviations

DSM-IV: Diagnostic and Statistical Manual of Mental Disorders, 4th version
ICD-10: International Classification of Diseases, 10th revision
NPR: Norwegian Patient Registry
TOP-study: Thematically Organized Psychosis study
